# Association Between Demographic and Socioeconomic Factors and Diagnosis of Advanced Non-small Cell Lung Cancer: An Analysis of the National Cancer Database

**DOI:** 10.7759/cureus.44351

**Published:** 2023-08-29

**Authors:** Kiana B Verplancke, Darby L Keirns, Kevin McMahon, Zachary A Creech, Gia Thinh Truong, Peter T Silberstein, Mary-Beth Dahl

**Affiliations:** 1 School of Medicine, Creighton University, Phoenix, USA; 2 School of Medicine, Creighton University, Omaha, USA; 3 Oncology, Creighton University School of Medicine, Omaha, USA

**Keywords:** health care disparities, stage at presentation, demographic factors, socio-economic factors, non small cell lung cancer

## Abstract

Introduction

Lung cancer is a prevalent and potentially lethal cancer. The stage at initial presentation for diagnosis predicts mortality and helps to guide treatment options. Thus, it is critical to determine what factors impact the stage of cancer at diagnosis. This study sought to determine if certain socioeconomic and demographic factors are associated with receiving an early (Stage 0-I) or advanced (Stage IV) diagnosis of non-small cell lung cancer (NSCLC).

Methods

Using the National Cancer Database (NCDB), 1,149,539 patients were identified as having an NCDB Analytic Stage Group diagnosis of Stage 0-I (early) versus Stage IV (advanced) NSCLC between 2004 and 2018. Patients with early and delayed diagnoses were compared based on specific characteristics including sex, race, ethnicity, number of comorbid conditions, insurance status, median annual income, level of education, geographic location, and reporting facility. Using IBM SPSS Statistics for Windows, Version 28 (Released 2021; IBM Corp., Armonk, New York, United States), the data underwent analysis using binary multivariate logistic regression, chi-square analyses, and one-way ANOVA.

Results

Factors associated with an advanced diagnosis of NSCLC include being male, Black, Native American, or Hispanic. Compared to patients with at least one comorbid condition, those without comorbid conditions are more likely to present with advanced disease. Patients with private insurance, Medicaid, Medicare, or other government insurance are all less likely to present with advanced-stage cancer than patients without insurance. Compared to patients in the lowest median household income quartile, those in the second and fourth quartiles are diagnosed earlier. Patients living in areas where a higher proportion of residents lack a high school diploma are more likely to present with advanced NSCLC. Additionally, living in the Midwest and Western United States and presenting to Community Cancer programs are associated with advanced disease at initial presentation.

Conclusions

Factors that were associated with the advanced presentation of NSCLC included being male, Black, Native American, or Hispanic, having a lack of comorbid conditions or insurance, earning a lower median annual income, and living in a zip code where a higher proportion of residents lack a high school diploma. Additionally, residing in the Midwest and Western United States and seeking care at Community Cancer programs were associated with advanced disease at initial presentation. Understanding that certain socioeconomic and demographic factors impact the stage at initial diagnosis of NSCLC can allow for targeted intervention strategies aimed at the most at-risk individuals, areas, and facilities.

## Introduction

Lung cancer ranks among the most frequently diagnosed cancers globally. In 2020, an estimated 2.21 million people worldwide received a new diagnosis of lung cancer [1]. According to statistics from the American Cancer Society (ACS), the most frequently diagnosed cancers for males in the United States in 2022 were prostate (27%), lung (12%), and colorectal (8%). For women, the most common diagnoses were breast (31%), lung (13%), and colorectal (8%). Regardless of gender, lung cancer exhibited a higher mortality rate than any other type of cancer. The ACS reports that more than 350 people die each day in the United States from lung cancer, accounting for more cancer-related deaths than breast, prostate, and pancreatic cancers combined. It is responsible for 250% more deaths than colorectal cancer, the second leading cause of cancer-related deaths [2].

Non-small cell lung cancer (NSCLC) accounts for 80-85% of all new lung cancer diagnoses [3]. Early diagnosis and treatment are crucial for improving outcomes and reducing mortality [4]. Cancer staging allows for the classification of disease severity at the time of diagnosis. The clinical stage represents an estimate of the cancer's extent, based on factors like tumor size and the presence of metastases. The stage at diagnosis plays a pivotal role in determining prognosis, influencing treatment plans, and impacting overall patient survival [5]. The globally accepted TNM staging model provides a standardized means of determining disease extent [6, 7]. In the TNM system, cancer is classified into a stage group ranging from zero to four, with higher numbers indicating greater size and spread of the disease [8]. For instance, stage I cancer indicates early-stage disease, confined to the organ where the cancer originated; stage IV cancer indicates advanced disease that has metastasized to distant sites in the body [7].

Currently, the five-year survival estimates for patients with NSCLC can vary dramatically, ranging from 90% for early-stage disease to less than 10% for advanced disease [9]. Such stark differences in survival rates underscore the critical importance of early diagnosis. Previous literature has explored the impact of demographic factors on cancer diagnosis and treatment. Factors like race, sex, age, and other socioeconomic disparities have been correlated with variations in cancer diagnosis, treatment, and mortality rates [10, 11]. Thus, identifying the socioeconomic and demographic variables that influence the stage of NSCLC at diagnosis can offer valuable insights into which patient populations are at the highest risk for an advanced diagnosis of NSCLC.

This study aimed to ascertain whether specific socioeconomic and demographic factors are associated with receiving an early (Stage 0-I) or advanced (Stage IV) diagnosis of NSCLC. We hypothesized that certain groups, defined by their socioeconomic and demographic characteristics, would be more likely to present with advanced-stage disease. This article was previously presented in poster form at the 2022 American Society of Clinical Oncology Annual Meeting in Chicago, Illinois, on June 6, 2022.

## Materials and methods

Data source

A retrospective review of NSCLC was carried out using data from the National Cancer Database (NCDB), a public database maintained by Commission on Cancer (CoC)-accredited facilities. Sponsored by the American Cancer Society and the American College of Surgeons, this nationwide database encompasses patient and facility data from all 50 states and captures approximately 70% of all cancer cases [12]. The NCDB contains de-identified patient statistics, including information on demographics, socioeconomic factors, tumor characteristics, and outcomes [13]. For this study, data were obtained from the NCDB on non-small cell lung cancer via a Participant User File (PUF) application. Upon approval of the PUF application, a data use agreement was signed to ensure the confidentiality of patients and appropriate handling of the dataset. The study received an exemption from the Creighton University Institutional Review Board (IRB) under submission number 2002623-01, as the NCDB uses de-identified data.

Study population

Patients were included in the study if they had a known stage at diagnosis and could be categorized as having either early or advanced-stage NSCLC based on the NCDB analytic stage group. Additionally, patients needed to have known demographic and socioeconomic variables for inclusion in each respective analysis. A total of 1,772,978 patients diagnosed with stage 0-IV NSCLC between 2004 and 2018 were identified. Of these, patients presenting with stage 0 and stage I at initial diagnosis were considered to have early-stage disease, accounting for 27.2% of identified patients. Those presenting with stage IV NSCLC at initial diagnosis were categorized as having an advanced presentation, making up 38% of the identified patient population. In total, 1,149,539 patients were included in the study with an NCDB analytic stage group diagnosis of either Stage 0-I (early) or Stage IV (advanced) NSCLC between 2004 and 2018. Meanwhile, 623,439 patients with stage II, III, occult lung cancer, or unknown AJCC staging at diagnosis were excluded from this study.

Outcomes of interest

The primary outcome of interest was the analytic stage at the initial diagnosis. If available, this value was the reported NCDB analytic stage group. This staging system classifies patients as having presented with Stage 0, Stage I, Stage II, Stage III, or Stage IV NSCLC on initial presentation. Following the completion of the initial analysis, an in-depth analysis of the intersectional impact of demographic variables was performed. To achieve this, multiple variables were grouped and their likelihood of receiving a later-stage diagnosis was compared.

Variables of interest

For analysis, variables of interest included certain socioeconomic and demographic factors at the patient and the facility level as recorded at the time of diagnosis. A patient-level factor included the patient’s primary insurance status, which was categorized as uninsured, private insurance, Medicaid, Medicare, or another form of government insurance. Education status was determined using a proxy measure based on the estimated proportion of adults who lived in the patient’s reported zip code without a high-school diploma. Zip-code statuses were defined as follows: 29% or more, 20% to 28.9%, 14% to 19.9%, and less than 14% of adults in the patient’s zip code without a high school diploma. The patient’s reported median annual income was stratified into quartiles based on equally proportioned income ranges among all United States zip codes: those who had a median annual income of less than $40,227 per year (quartile one), those who made between $40,228 and $50,353 (quartile two), those who made between $50,354 and $63,332 (quartile three), and those who had a median income of $63,333 or more (quartile four) a year. In addition, the Charlson/Deyo score was considered. The Charlson/Deyo score ranges from zero to three, with zero representing no comorbidities, one representing single comorbidity, two representing two comorbidities or a single comorbidity with a weight of two, and three meaning the patient has significant comorbidities [14]. Each patient’s age, biological sex, race, and ethnicity were also considered. 

At the facility level, a variable of interest included the geographic region where the facility was located. Geographic regions were divided into Northeast, South, Midwest, and West based on U.S. Census Division designations. Facility type was also considered, with four classifications: community cancer programs, comprehensive community cancer programs, academic/research programs, and integrated network cancer programs. Each location was defined according to the Commission on Cancer Accreditation Program.

Statistical analysis

Using IBM SPSS Statistics for Windows, Version 28 (Released 2021; IBM Corp., Armonk, New York, United States), patients with early and advanced diagnoses were compared to each other based on socioeconomic and demographic variables. Descriptive statistics were used to summarize the characteristics of the study population. Results were initially analyzed with chi-square analyses. A multivariate binary logistic regression to examine independent associations between the initial stage at diagnosis and demographic and socioeconomic characteristics was also conducted. Any data with missing information on stage or demographic/socioeconomic variables was excluded from the analysis. A 95% confidence interval (CI) was utilized for this analysis.

## Results

In this study, statistically significant differences in stage at initial presentation were identified among patients with NSCLC. Table 1 summarizes the descriptive statistics of patients who presented with either early or advanced-stage NSCLC. Among the patient categories, a higher proportion of males, as well as Black, Native American, and Hispanic individuals, were diagnosed at a later stage. As for insurance status, a greater number of uninsured patients, or those with private insurance or Medicaid, presented with advanced disease. A majority of patients with median annual incomes falling within quartile one (less than $47,227) and quartile two ($40,228 to $50,353) exhibited advanced rather than early-stage disease upon initial presentation. Additionally, a higher number of patients who presented to community cancer programs and comprehensive community cancer programs were diagnosed with advanced disease. Geographically, more patients residing in the South, Midwest, and West presented with advanced disease compared to those presenting with early disease. Lastly, it was determined that patients diagnosed with early-stage disease presented on average 2.22 years later than those diagnosed with advanced NSCLC. The mean age at diagnosis for patients who presented early was 69.74, while for those who presented later, it was 67.52.

**Table 1:** Patient and Facility Descriptive Statistics for Early and Advanced-Stage NSCLC Cases From the National Cancer Database

Demographic/Socioeconomic Factor	Variable	Number of Patients With Stage Group 0-1	Number of Patients With Stage Group IV	Overall Number of Patients in the Group
Sample size	n/a	482,448 (42%)	667,091 (58%)	1,149,539 (100%)
Average age (years)	n/a	69.74	67.52	n/a
Sex	Male	221,757 (46.00%)	364,846 (54.70%)	586,603 (51.00%)
	Female	260,691 (54.00%)	302,245 (45.30%)	562,936 (49.00%)
Race	White	424,756 (88.60%)	555,655 (83.90%)	980,411 (85.90%)
	Black	41,154 (8.60%)	81,178 (12.30%)	122,332 (10.7%)
	Native American	1,182 (0.20%)	1,809 (0.3%)	2,991 (0.30%)
	Other	12,103 (2.50%)	23,445 (3.50%)	35,548 (3.10%)
Ethnicity	Non-Hispanic	447,013 (99.7%)	610,236 (99.5%)	1,057,249 (99.6%)
	Hispanic	1,272 (0.3%)	3,174 (0.5%)	4,446 (0.4%)
Charlson-Deyo comorbidity index	0	256,428 (53.2%)	414,169 (62.10%)	670,597 (58.30%)
	1	144,642 (30%)	163,107 (24.5%)	307,749 (26.8%)
	2	55,215 (11.4%)	59,042 (8.9%)	114,257 (9.9%)
	>3	26,163 (5.4%)	30,773 (4.6%)	56,936 (5%)
Insurance status	Not Insured	6,227 (1.3%)	26,760 (4%)	32,987 (2.9%)
	Private insurance/managed care	116,095 (24.1%)	186,563 (28%)	302,658 (26.3%)
	Medicaid	20,672 (4.3%)	51,655 (7.7%)	72,327 (6.3%)
	Medicare	324,507 (67.3%)	379,516 (56.9%)	704,023 (61.2%)
	Other government	7,729 (1.6%)	9,749 (1.5%)	17,478 (1.5%)
	Insurance status unknown	7,218 (1.5%)	12,848 (1.9%)	20,066 (1.7%)
Median annual income	Less than $40,227	84,681 (19.40%)	135,126 (21.6%)	219,807 (20.7%)
	$40,228-$50,353	100,721 (23%)	148,276 (23.7%)	248,997 (23.4%)
	$50,354-$63,332	103,804 (23.7%)	146,834 (23.5%)	250,638 (23.6%)
	$63,333 or more	148,195 (33.9%)	195,507 (31.2%)	343,702 (32.2%)
Education (percent in zip code w/o high-school diploma)	29.0% or more	70,000 (16.2%)	115,905 (18.8%)	185,905 (17.7%)
	20.0%-28.9%	105,524 (24.5%)	155,867 (25.2%)	261,391 (24.9%)
	14.0%-19.9%%	107,541 (24.9%)	150,272 (24.3%)	257,813 (24.6%)
	Less than 14.0%	148,175 (34.4%)	195,863 (31.7%)	344,038 (32.8%)
Facility type	Community Cancer Program	26,131 (5.4%)	57,246 (8.6%)	83,377 (7.3%)
	Comprehensive Community Cancer Program	191,775 (40%)	274,409 (41.4%)	466,184 (40.8%)
	Academic/Research Program	164,118 (34.2%)	195,757 (29.6%)	359,875 (31.5%)
	Integrated Network Cancer Program	97,833 (20.4%)	134,663 (20.3%)	232,496 (20.4%)
Geographic location	Northeast	219,779 (45.8%)	288,334 (43.6%)	508,113 (44.5%)
	South	71,895 (15%)	104,526 (15.8%)	176,421 (15.4%)
	Midwest	125,988 (26.3%)	178,136 (26.9%)	304,124 (26.6%)
	West	62,195 (13%)	91,079 (13.8%)	153,274 (13.4%)

Based on these findings, a multivariate binary logistic regression analysis was performed, and the findings are presented in Table 2. At the patient level, females were 70% less likely to be diagnosed with advanced disease compared to males. Black and Native American patients were more likely to present with advanced disease in comparison to White patients. Hispanic patients were 49.3% more likely to present with advanced disease than non-Hispanic patients. Patients with at least one comorbidity (CCI score ≥ 1) were less likely to present with advanced disease. Uninsured patients were more likely to be diagnosed with advanced disease than patients with some form of insurance. Specifically, those with private insurance were 40% less likely, those with Medicaid were 58% less likely, those with Medicare were 31% less likely, and those with some other form of government insurance were 30% less likely to be diagnosed with advanced-stage disease. Compared to patients with a median annual income in quartile one (less than $47,227), those in quartiles two ($40,228 to $50,353) and four (more than $63,332) exhibited a 98% and 96% lower likelihood, respectively, of presenting with advanced-stage disease. Notably, there was no statistically significant difference in the stage at initial presentation between patients with median annual incomes in quartile one (less than $47,227) and those in quartile three ($50,354 to $63,332). Patients residing in zip codes where 29% or more of its residents lacked a high school diploma were more likely to present with advanced NSCLC compared to patients in areas where less than 28.9% of its residents did not graduate from high school.

At the facility level, patients who presented to community cancer programs were more likely to have delayed initial presentation. Specifically, those who presented to comprehensive community cancer programs, academic/research programs, and integrated network cancer programs were 66.9%, 52%, and 65.5% less likely to present with advanced disease, respectively. Geographically, patients in the Midwest and Western United States were 10% and 5% more likely to present with advanced disease than patients living in the Northeast United States, respectively. No significant difference was found between patient populations living in the Northeast and Southern United States.

**Table 2:** Multivariate Logistic Regression Results for Odds of Advanced Stage Non-small Cell Lung Cancer at Initial Presentation in Patients Diagnosed From The National Cancer Database (2004-2018) Variables in rows without numbers are the reference variables.

Factor	Variable	Odds Ratio	Confidence Interval: Lower Bound	Confidence Interval: Upper Bound	P-Value
Sex	Male				
	Female	0.702	0.697	0.708	≤0.001
Race	White				
	Black	1.428	1.407	1.449	≤0.001
	American Indian	1.137	1.046	1.235	≤0.002
	Other	1.36	1.325	1.395	≤0.001
Ethnicity	Non-Hispanic				
	Hispanic	1.493	1.389	1.605	≤0.001
Charlson-Deyo comorbidity index	0				
	1	0.704	0.698	0.711	≤0.001
	2	0.675	0.666	0.685	≤0.001
	>3	0.73	0.716	0.744	≤0.001
Insurance status	Not insured				
	Private insurance/managed care	0.408	0.395	0.421	≤0.001
	Medicaid	0.585	0.564	0.607	≤0.001
	Medicare	0.318	0.308	0.329	≤0.001
	Other government	0.3	0.287	0.314	≤0.001
	Insurance status unknown	0.445	0.425	0.466	≤0.001
Median household income	Less than $40,227				
	$40,228-$50,353	0.978	0.965	0.992	≤0.001
	$50,354-$63,332	0.988	0.973	1.003	0.111
	$63,333 or more	0.959	0.943	0.976	≤0.001
Education (percent in zip code w/o high-school diploma)	29.0% or more				
	20.0%-28.9%	0.961	0.948	0.974	≤0.001
	14.0%-19.9%	0.941	0.926	0.955	≤0.001
	Less than 14.0%	0.934	0.917	0.951	≤0.001
Facility type	Community Cancer Program				
	Comprehensive Community Cancer Program	0.669	0.657	0.68	≤0.001
	Academic/Research Program	0.52	0.511	0.529	≤0.001
	Integrated Network Cancer Program	0.655	0.643	0.668	≤0.001
Geographic location	Northeast				
	South	0.1002	0.989	1.015	0.773
	Midwest	1.1	1.089	1.112	≤0.001
	West	1.052	1.039	1.066	≤0.001

Considering these results, subgroups were created to further compare the impact of intersectional demographics on delayed presentation. Findings from a second multivariate logistic regression analysis are presented in Table 3. When comparing a favorable scenario (female, White, living in a zip code where at least 29% of residents have a high school diploma, having private insurance, and a median annual income of $63,333 or more) with a less favorable scenario (male, Black, living in a zip code where less than 14% of residents have a high school diploma, being uninsured, and having a median annual income of $47,227 or less) patients with the less favorable factors were 529% more likely to be diagnosed with advanced stage NSCLC cancer than those with more favorable factors. Secondly, a comparison was made between Black patients who otherwise had variables typically associated with early presentation (female, Black, living in a zip code where at least 29% of residents have a high school diploma, having private insurance, and a median annual income of $63,333 or more) and White patients who otherwise had variables associated with delayed presentation (male, White, living in a zip code where less than 14% of residents have a high school diploma, being uninsured, and having a median annual income of $47,227 or less). Despite this change, it was determined that Black patients with otherwise more advantageous variables were 126% more likely than White patients with otherwise less advantageous variables to present with advanced NSCLC.

Next, the impact of being Hispanic on stage at the initial presentation was evaluated. Initially, patients who had less favorable factors and who were Hispanic (male, Hispanic, living in a zip code where less than 14% of residents have a high school diploma, being uninsured, and having a median annual income of $47,227 or less) and patients with more favorable patient factors and were not Hispanic (female, non-Hispanic, living in a zip code where at least 29% of residents have a high school diploma, having private insurance, and a median annual income of $63,333 or more) were compared. Patients who were Hispanic and had less favorable factors were 1,395% more likely to be diagnosed with advanced NSCLC than those with more favorable factors and who were not Hispanic. A second comparison between patients who were not Hispanic with otherwise less favorable patient factors (male, non-Hispanic, living in a zip code where less than 14% of residents have a high school diploma, being uninsured, and having a median annual income of $47,227 or less) and Hispanic patients with otherwise the more favorable patient factors (female, Hispanic, living in a zip code where at least 29% of residents have a high school diploma, having private insurance, and a median annual income of $63,333 or more) was also made. It was determined that Hispanic patients with otherwise favorable variables were 333% more likely to present with advanced disease than non-Hispanic patients with otherwise unfavorable patient factors.

**Table 3:** Multivariate Logistic Regression Results for Odds of Advanced Stage Non-small Cell Lung Cancer at Initial Presentation in Black and Hispanic Patients in Comparison to White and Non-Hispanic Patients From the National Cancer Database (2004-2018) Variables in rows without numbers are the reference variables.

Grouped Variables	Odds Ratio	Confidence Interval: Lower Bound	Confidence Interval: Upper Bound	P-Value
Most favorable scenario				
Least favorable scenario	5.294	4.536	6.179	≤0.001
Less favorable variables and not Hispanic				
Most favorable variables and Hispanic	3.325	1.778	6.219	≤0.001
Most favorable variables and not Hispanic				
Less favorable variables and Hispanic	13.952	5.096	8.916	≤0.001
Less favorable variables and White				
Most favorable variables and Black	1.257	1.101	1.436	≤0.001

## Discussion

The goal of the present study was to examine the socioeconomic and demographic differences between patients who at initial presentation were diagnosed with early or advanced-stage NSCLC. Findings from this study revealed that significant socioeconomic and demographic differences at the patient and facility levels were associated with significantly increased odds of advanced initial presentation and diagnosis. This finding contributes to the understanding of inequities in healthcare. 

According to this study, patients with lower socioeconomic status or who belonged to minority groups had increased odds of receiving a delayed NSCLC diagnosis. Lower education status, as determined by the percentage of residents without a high school diploma in a patient’s reported zip code, also contributed to advanced NSCLC diagnosis. Therefore, when advocating for patients, health literacy and awareness campaigns should be directed towards educating those from lower education backgrounds. Education should emphasize the importance of regular screening for higher-risk patients, such as tobacco users, and provide details regarding the signs and symptoms of lung cancer [15, 16]. Lack of health insurance also contributed to increased odds of advanced presentation, stressing the role of health insurance in timely diagnosis. Therefore, expanding health insurance coverage and implementing policies to ensure access to preventative health care services are important steps to improve patient outcomes.

Lower median annual income seems to play a role in presenting with advanced disease. However, when comparing patients whose median annual incomes lie in quartile one with those who have incomes that lie in quartile three, there was no significant difference in stage at the initial presentation. Therefore, further research is needed to determine exactly how much income impacts the stage at initial presentation and diagnosis. Nevertheless, when comparing patients whose median annual income was in quartile one with those in quartile four, there was a significant difference in the odds of advanced presentation. Suggesting a role in advocating for policies that provide affordable and accessible healthcare. The association between a lack of comorbid conditions and a delay in NSCLC diagnosis could suggest that patients being diagnosed later do not have easy access to healthcare services and therefore are unaware of the underlying conditions to report. However, further research is needed to ascertain an explanation for these findings.

Furthermore, geographic location and primary reporting facility type also influenced the stage of diagnosis of NSCLC. Patients residing in the Midwest and Western United States were more likely to be diagnosed with advanced disease in comparison to patients in the Northeast. When looking at the geographic location of National Cancer Institute centers the majority are in the Northeast, with fewer in the Midwest and the West [17]. The lack of cancer centers in certain regions is a possible explanation for this finding. In addition, people were more likely to present with advanced-stage disease if they presented to community cancer programs. Although, the explanation for this finding cannot be determined without further research. Investigating the acuity of each patient’s condition and determining whether specific facility types receive more complex patients with more medical needs could provide insight into how facility type influences the timing of cancer detection.

A key finding is the significant association between delayed diagnosis for Black and Hispanic patients in comparison to their White and non-Hispanic counterparts. Even when all other patient factors are in favor of earlier diagnosis in Black and Hispanic patients and all other patient factors are in favor of a delayed diagnosis in White and non-Hispanic individuals. The findings in this study are consistent with similar cancer-related research. For instance, in previous research, such as in findings reported by Efird et al., Black patients were more likely than White patients to have an advanced stage of cancer at initial presentation despite having the same insurance [18]. Additionally, in a previous review published by the Institute of Medicine, socioeconomic factors, such as those described above, are associated with risk factors for cancer, such as tobacco use, income, education, health insurance coverage, and poor nutrition [16]. This suggests that the explanation for our findings is multifactorial and part of a legacy of social inequities in the United States.

Overall, this study underscores the importance of continued patient advocacy and education. Addressing the socioeconomic and demographic disparities identified by this research requires a multifaceted approach. Health initiatives and policies should focus on emphasizing the importance of early detection and screening for lung cancer, especially in the higher-risk populations identified by this study. In addition, cancer screening programs should be implemented across all geographic regions of the United States with a particular focus on areas that would target underserved populations, such as communities with lower educational status. These initiatives could lead to the earlier identification of NSCLC cases and better patient outcomes.

Moreover, the consideration of not only clinical factors but also a patient’s socioeconomic status and individual barriers to health care is a key component of earlier detection of NSCLC. Conducting qualitative research to explore patient viewpoints regarding obstacles to early diagnosis or interventions emerges as an important next step. One that has the potential to aid in reducing health disparities in the diagnosis of NSCLC. In addition, calling for teamwork among health care providers, the community, and government officials is essential to the development of targeted interventions to address the needs of the vulnerable populations identified by this research.

Study limitations

Limitations to this study include that this data is based on retrospective data obtained from the NCDB. While the NCDB accounts for roughly 70% of new cancer diagnoses in the United States, data may not be representative of the entire population [19]. In addition, if cancer facilities do not participate in the NCDB registry their data is unaccounted for [20].

## Conclusions

In conclusion, significant differences between the stage at the initial presentation and certain socioeconomic and demographic factors were identified. Patient factors that were associated with a delayed initial presentation in patients diagnosed with NSCLC included male sex, Black or Native American race, being Hispanic, having no comorbid conditions, being uninsured, having a lower median annual income, and residing in a zip code with a lower proportion of residents holding a high school diploma. Facility factors that were associated with delayed patient presentation included presenting to a Community Cancer Program and presenting to a hospital in the Midwest and Western United States. Addressing these disparities requires focused interventions that seek to improve healthcare access, coverage, and health literacy, and address biases within the healthcare system. Beginning to discuss and implement strategies to target these populations can allow us to achieve more equitable outcomes for all patients affected by NSCLC.
